# Effectiveness of the Tailored, Early Comprehensive Rehabilitation Program (t-ECRP) based on ERAS in improving the physical function recovery for patients following minimally invasive esophagectomy: a prospective randomized controlled trial

**DOI:** 10.1007/s00520-022-06924-8

**Published:** 2022-02-22

**Authors:** Funa Yang, Lijuan Li, Yanzhi Mi, Limin Zou, Xiaofei Chu, Aiying Sun, Haibo Sun, Xianben Liu, Xiaoxia Xu

**Affiliations:** 1 Nursing Department, The Affiliated Cancer Hospital of Zhengzhou University, Henan Cancer Hospital, Zhengzhou, 450008 China; 2 Department of Thoracic Surgery, The Affiliated Cancer Hospital of Zhengzhou University, Henan Cancer Hospital, Zhengzhou, 450000 China

**Keywords:** Esophageal cancer, Minimally invasive esophagectomy, Bowel function, Physical function, Rehabilitation

## Abstract

**Background:**

Perioperative rehabilitation management is essential to enhanced recovery after surgery (ERAS). Limited reports, however, have focused on quantitative, detailed early activity plans for patients receiving minimally invasive esophagectomy (MIE). The purpose of this research was to estimate the effectiveness of the Tailored, Early Comprehensive Rehabilitation Program (t-ECRP) based on ERAS in the recovery of bowel and physical functions for patients undergoing MIE.

**Methods:**

In this single-blind, 2-arm, parallel-group, randomized pilot clinical trial, patients admitted to the Affiliated Cancer Hospital of Zhengzhou University from June 2019 to February 2020 were selected and randomly assigned to an intervention group (IG) or a control group (CG). The participants in the IG received medical care based on the t-ECRP strategy during perioperative period, and participants in the CG received routine care. The recovery of bowel and physical functions, readiness for hospital discharge (RHD), and postoperative hospital stay were evaluated on the day of discharge.

**Results:**

Two hundred and fifteen cases with esophageal cancer (EC) were enrolled and randomized to the IG (*n* = 107) or CG (*n* = 108). The mean age was 62.58 years (SD 9.07) and 71.16% were male. For EC, 53.49% were mid-location cancers and 79.07% were classified as pathological stage II and III cancers. There were no significant differences between the two groups in terms of demographic and clinical characteristics and baseline physical functions. Participants in the IG group presented significantly shorter lengths of time to first flatus (*P* < 0.001), first postoperative bowel movement (*P* = 0.024), and for up and go test (*P* < 0.001), and lower scores of frailty (*P* < 0.001). The analysis also showed that participants in the IG had higher scores of RHD and shorter lengths of postoperative stay than in the CG (*P* < 0.05).

**Conclusions:**

The t-ECRP appears to improve bowel and physical function recovery, ameliorate RHD, and shorten postoperative hospital stay for patients undergoing MIE. Clinicians should consider prescribing quantitative, detailed, and individualized early activity plans for these patients.

**Trial registration:**

ClinicalTrials.gov (Identifier: NCT01998230)

## Introduction

Esophageal cancer (EC), a malignant tumor that occurs in the epithelial tissue of the esophagus, is the eighth most common cancer and the sixth most common cause of death overall on the global burden of cancer worldwide [1]. In China, the latest epidemiological survey showed that around 145,700 new cases and 188,100 deaths of EC occurred in 2015, which were higher than the average level worldwide [2]. Surgery is still the standard treatment for resectable EC with unacceptable morbidity and mortality rates. A global review of high-volume hospitals performing esophagectomy showed overall morbidity of 59% and 30-day mortality of 2.4% [3].

Many new strategies and technologies attempt to reduce complications and promote fast recovery, such as minimally invasive esophagectomy (MIE) and the concept of enhanced recovery after surgery (ERAS). ERAS was described first in 1997 by Henrik Kehlet, which has been widely applied to reduce the surgical stress response, postoperative medical complications, and hospital stay, and improve recovery after surgery [4–7]. ERAS was initially applied in colorectal cancer and subsequently expanded to orthopedics, gynecologic, urology, and colorectal [8, 9]. The guidelines for perioperative care in esophagectomy were published by the ERAS Society in 2019, which provided standard norms for perioperative ERAS care protocol of EC [7].

The key determinant in evaluating the success of ERAS is whether the patients’ functional activities can quickly recover to an acceptable level after surgery [7]. Research indicated that postoperative mobilization should start on the day of surgery, and gradually increase the amount of activities to achieve predetermined goals [10]. Long-term bed rest after surgery increases the risk of complications, such as venous thromboembolism, muscle loss, insulin resistance, and pulmonary complications [11, 12]. Patients with EC are often accompanied by malnutrition, frailty, pain, and drainage pipes, which make it more difficult for patients to rehabilitate early and adequately. Although some non-randomized studies concluded that early mobilization might hasten functional recovery after surgery, the evidence on the timing and nature of mobilization is lacking [7].

Physical function is associated with postoperative mortality in patients with cancer [13]. Results from longitudinal studies showed that the physical function levels of patients undergoing esophagectomy tended to be lower compared with their preoperative levels [14]. Several measures could evaluate physical functions available, such as fatigue level and timed get up and go test. Previous research has shown that a planned exercise program could improve physical function and reduce fatigue for cancer survivors. However, the majority of intervention studies have focused on colorectal and breast cancer [15], and few trials focused on EC survivors after MIE.

It is well known that the implementation of ERAS can speed up patient turnover, which means less time is required for hospital discharge. Readiness for hospital discharge (RHD) is a transitional outcome in the continuum of care from hospital to home [16]. Inadequate RHD is associated with adverse outcomes, such as complications occurrence and unplanned readmission, and a good transition can promote recovery and achieve a better outcome [17]. Therefore, it is essential to pay attention to and improve the RHD of patients during their hospitalization.

ERAS can effectively shorten the lengths of time to first postoperative flatus and bowel movement, and improve the physical functions of patients [18, 19]. However, ERAS combines a series of evidence-based perioperative optimization measures, including preoperative prehabilitation, early ambulation, early eating, and pain control. Therefore, the relationship between early activity management and postoperative intestinal and physical function recovery is still unknown.

Overall, although perioperative rehabilitation management after esophagectomy is crucial, few studies have focused on the formulation of early postoperative rehabilitation programs. An early, standardized, quantitative, and comprehensive rehabilitation intervention program tailored to individual patients and based on ERAS is thus urgently needed. In this study, we hypothesized that the Tailored, Early Comprehensive Rehabilitation Program (t-ECRP) based on ERAS might improve bowel and physical function recovery for patients after MIE. This randomized controlled clinical trial was conducted to evaluate the role of t-ECRP in improving recovery outcomes of EC patients after surgery and thus could provide a reference for clinical work.

## Materials and methods

### Study design and setting

This single-blind, 2-arm, parallel-group, randomized pilot clinical trial was conducted at the Affiliated Cancer Hospital of Zhengzhou University, Zhengzhou, China. With the help of randomization codes produced by means of the PROC PLAN of the SAS system, patients with EC undergoing MIE were randomly divided into intervention group (IG) and control group (CG) with a 1:1 assignment ratio. Researchers involved in the formulation and implementation of intervention programs were informed about the allocated intervention. However, research assessors, data management staff, and all patients were blinded to the intervention. Furthermore, research subjects would be placed into different wards to avoid mutual interference among patients. This study obtained written informed consent from all subjects or their families before the trial. In addition, the principles of the Helsinki Declaration were strictly followed. This study was approved by the ethics committee of the local medical ethics committee (2014xjs4), and the protocol was registered in the ClinicalTrials.gov (NCT01998230) database.

### Study participants

The study was performed between June 2019 and February 2020 at the Department of Thoracic Surgery of the Affiliated Cancer Hospital of Zhengzhou University. All patients undergoing MIE surgery were recruited according to the following criteria. Eligibility criteria are as follows: (a) histologically proven EC and selected for MIE; (b) age ≤ 75 years; (c) volunteer to this research; and (d) signed written informed consent. Exclusion criteria included (a) previous severe lung, brain, and heart organic diseases, and bone and joint disorders; (b) emergency surgery; (c) serious postoperative complications such as anastomotic leakage; and (d) inability to perform language communication or text understanding.

### t-ECRP procedures

Participants in the IG received the t-ECRP from admission to discharge. A t-ECRP team was assembled before the intervention, including two thoracic surgeons, four nurses, and one physiotherapist. A comprehensive evaluation was conducted before intervention, such as disease conditions, cardiopulmonary function, disease cognition, self-disease management ability, and social support. Then, a tailored ECRP practical target was developed with the joint participation of patients and the research team.

According to the treatment procedures, the t-ECRP was designed to be consisting of three main stages based on the concept of ERAS: (1) preoperative prehabilitation, which was defined as the duration from admission to the day before surgery; (2) the day of surgery; and (3) postoperative rehabilitation, which was defined as the duration from the first day after surgery to discharge. The procedure of early comprehensive rehabilitation program was as follows and is shown in Table 1.

- Stage I: Participants were required to perform steps climbing training (SCT) and inspiratory muscle training (IMT) in the rehabilitation training room under the guidance of professionals. At program commencement, all participants received one face-to-face instructional session. The SCT was performed 3 to 5 times per day, 10 min each time. During the SCT, the step height was set at 15 cm training speed controlled at 20 ~ 40 steps/min, and individualized training intensity would be adjusted in time by a physiotherapist after physical conditions evaluation. The IMT was carried out using a handheld tapered flow resistive inspiratory loading device (K3, POWER breathe ®) with a frequency of 6 to 8 times per day, 10 min each time. At the beginning of training, 60% of the maximal inspiratory pressure was applied. The exercise intensity was adjusted in time by the physiotherapist according to participant-reported rate of perceived exertion.
- Stage II: On the day of surgery, participants began to exercise on the bed after waking up from anesthesia. The whole training included toe flexion and extension, ankle joint and knee joint movement, leg muscle isometric contraction, and hips lifting off the bed, and was repeated 2–3 times on the day of surgery led by a nurse.
- Stage III: Participants were encouraged to get out of bed on POD (postoperative day) 1 for 4–6 times following the “5–3-1 methods”: sitting on the bed for 5 min, standing on the bed for 3 min, and moving the legs and feet for 1 min under the help and guidance of nurses. Then, participants started to walk on POD 2 in the ward corridor, and an individualized daily walking plan was tailored based on participants’ physical status, as well as the advice from the thoracic surgeon and physiotherapist. For example, on POD 2–3, participants were recommended to walk 6 times per day with a target distance of 500–1000 m; 8 times walking per day with 1000–1500 m on POD 4–5, and more than 8 times per day with 2000 m from POD 6 to discharge. The trained nurses would motivate and promote patients to carry out a daily walking plan, and an appropriate adjustment of the walking plan was made if necessary. Besides, preoperative IMT was required and carried out under the supervision of a physiotherapist.

**Table 1:** Procedure of early comprehensive rehabilitation program after MIE for EC of the intervention group

Stage	Time	Items	Frequency
Stage I	Preoperative	SCT	• 3 to 5 times per day • 10 min each time
		IMT	• 6 to 8 times per day • 10 min each time
Stage II	The day of surgery	Exercise on the bed	• 2–3 times, led by nurse
		IMT	• Same as above
Stage III	POD 1	Bedside activity	• 4 to 6 times per day(1–2 times in the morning, 3–4 times in the afternoon)
		IMT	• Same as above
	POD 2–3	Walking	• 6 to 8 times per day • 5–10 min each time • total target quantity: 500–1000 m
		IMT	• Same as above
	POD 4–5	Walking	• ≥ 8 times per day • 15–20 min each time • total target quantity: 1000–1500 m
		IMT	• Same as above
	POD 6 to Discharged	Walking	• ≥ 8 times per day • 15–20 min each time • total target quantity: above 2000 m
		IMT	• Same as above

*SCT*, stair climbing training; *IMT*, inspiratory muscle training; *POD*, postoperative day

Throughout the intervention process, the guidance and supervision of medical staff were essential, especially when patients began to perform SCT and got out of bed for the first time. Rehabilitation activities should stop immediately if patients suffered from arrhythmia, chest tightness, suffocation, and other discomforts, and the rehabilitation plan would restart only following an evaluation and treatment process by the t-ECRP team. The times and amount of participants’ daily activities were recorded in predesigned tables.

### Control group

Patients in CG received usual nursing measures after MIE, including conventional postoperative feeding, pain management, provision of a safe and comfortable environment, wound care, diet guidance, medication care, psychological counseling, and regular postoperative rehabilitation exercises. The pulmonary rehabilitation and physical activity were conducted by nurses according to the procedures for routine postoperative care.

### Primary endpoints

Bowel function recovery was measured by the time to first postoperative flatus and bowel movement. Physical function was assessed by the timed up and go test and frailty scores. In the timed up and go test (TUGT), the times taken for participants to finish the following sequential movements were recorded: stand from a chair, walk 3 m, turn around, walk back to the chair, and sit down [20]. The TUGT was performed twice, and the average value was used. The frailty score was developed by Fried and colleagues [21], whose criteria comprise five components: exhaustion, unintentional weight loss, slowness, weak muscle strength, and low physical activity. For the five frailty criteria, 1 score would be given for each criterion when it was met. The total scores ranged between 0 and 5, and participants were classified as either robustness states (0 score), pre-frailty (1 or 2 scores), or frailty (3 or more scores) [22].

### Secondary endpoints

Readiness for hospital discharge (RHD) and postoperative hospital stay were the secondary endpoints. RHD could be used to capture patients’ perceptions of readiness for discharge. The RHD questionnaire was developed by Weiss et al. in 2006 [23] and had been translated and revised into a Chinese version by Taiwanese scholars [24]. This Chinese version scale consists of 12 items and 3 dimensions, covering physical status, adaptive ability, and expected support. The score range of each item is from 0 to 10, and higher scores indicate better readiness. The overall Cronbach’s *α* coefficient of the scale was 0.89 [24], confirming its validity.

All data were collected by trained nurses using Microsoft® Excel. The validity of forms was checked and entered into the data management Excel by trained study staff. A consistency check was conducted, and mistakes were corrected by retrieving the original records if inconsistencies were identified. Each participant had a unique identification code which was used to track all of the individual’s relevant documentation forms.

### Sample size calculation

The sample size was calculated based on the primary outcome—the time to first flatus after surgery. Previously published results [25] showed that the mean lengths of time to first flatus in the IG and CG were 2.6 days and 3.4 days, respectively, and the standard deviation was 1.7 days. A sample size of 72 would give an 80.08% power to reject the null hypothesis of equal means when the population means difference is 0.8 (*μ*1–*μ*2 = 3.4–2.60), with a standard deviation for both groups of 1.7 and a significant level (alpha) of 0.05, using a two-sided two-sample equal-variance *t*-test by PASS 15.0 software. Given a 20% allowance for attrition, the sample size was increased to 180 patients (90 participants per group) at baseline.

### Statistical analysis

Descriptive statistics was used for demographic and clinical data at baseline. Continuous variables were presented as means ± SD and compared using the unpaired *t*-test. Categorical or ranked variables were presented as frequency (%)and analyzed using the *χ*^2^. *P* < 0.05 was considered statistically significant. The statistical analysis was performed using SAS 9.4 (SAS Institute Inc., Kerry, USA).

## Results

### Participant recruitment flow

**Fig. 1:**
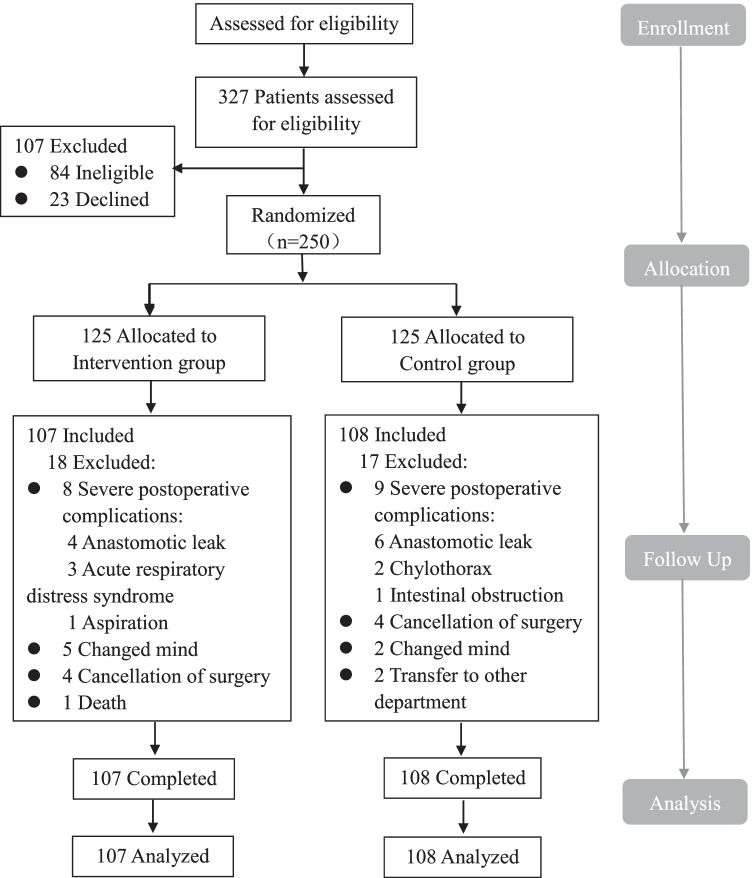
Consort diagram for the study

Three hundred and twenty-seven potential participants were recruited from June 2019 to February 2020, of whom 250 (76.45%) were included, and randomized into two groups to receive routine care or t-ECRP. During the research, 35 patients were excluded (18 patients in IG and 17 patients in CG), and 215 patients were included in the final analyses (IG, *n* = 107; CG, *n* = 108). The main reasons for patient withdrawal were severe postoperative complications, cancelation of surgery, and changed mind. The detailed selection process of the participants is shown in Fig. 1.

### Baseline characteristics

At baseline, all patients underwent a preoperative assessment on the day of admission, and data about sociodemographic details, medical history, and comorbidities were collected. A total of 215 patients (male: 153; female: 72) with a mean age of 62.58 years were recruited in this study. The majority of subjects were living with family members (198/215, 92.09%), and approximately half of subjects had middle location tumors (115/215, 53.49%). Most of the tumors were pathologically classified as stages II and III tumors (170/215, 79.07%), and 20 (9.30%) participants experienced recurrent temporary nerve paralysis. Demographic and clinical characteristics were similar between the intervention and control groups with no statistical difference, shown in Table 2.

**Table 2:** Demographic and clinical characteristics of patients with MIE in intervention and control groups

Variable	Intervention group (*n* = 107)	Control group (*n* = 108)	Statistics	*P* value
Age, y	63.09 ± 8.98	61.14 ± 10.02	1.50^a^	0.135
Gender			1.56^b^	0.212
Male	72	81		
Female	35	27		
Marital status			1.34^b^	0.512
Married	98	103		
Divorced	6	3		
Widowed	3	2		
Living situation				
Living alone	12	5	3.20^b^	0.074
Living with family	95	103		
Occupational status			3.01^b^	0.08
Employed	39	52		
Unemployed or retired	68	56		
Operation time, h	5.11 ± 0.63	4.97 ± 0.75	1.90^a^	0.058
Location of tumor			5.46^b^	0.065
Upper	18	16		
Middle	64	51		
Lower	25	41		
Pathological stage^*^			7.46^b^	0.059
0	3	2		
I	18	22		
II	30	46		
III	56	38		
Recurrent nerve paralysis			0.62^b^	0.734
No	94	98		
Yes	11	9		
Uncertain	2	1		

*MIE*, minimally invasive esophagectomy

^*^According to the 8th edition TNM staging standard of esophageal cancer by the Union for International Cancer Control

^a^Independent *t*-test

^b^*χ*^2^ test

### Efficacy of the t-ECRP

The research outcomes were measured again on the day of discharge after t-ECRP intervention or routine care, which usually was on the 7–9th day postoperative. The primary outcomes about bowel function and physical function recovery are outlined in Tables 3 and 4, respectively. Compared with the CG, patients in the IG presented significantly shorter lengths of time to first flatus (3.24 days vs. 4.19 days, *P* < 0.001) and first bowel movement (4.55 days vs. 5.38 days, *P* = 0.024), shown in Table 3.

**Table 3:** The bowel function recovery of patients in intervention and control groups after MIE

Variable	Intervention group (*n* = 107)	Control group (*n* = 108)	*t* value	*P* value
Time to first flatus (d)	3.24 ± 1.11	4.19 ± 1.67	− 4.92	< 0.001
Time to first bowel movement (d)	4.55 ± 2.34	5.38 ± 2.98	− 2.27	0.024

*MIE*, minimally invasive esophagectomy

**Table 4:** The physical function recovery of patients with MIE at pre-intervention and post-intervention of intervention and control groups

Variable	Pre-intervention		Post-intervention	
	Time of TUGT (s)	Frailty score	Time of TUGT (s)	Frailty score
Intervention group (*n* = 107)	9.01 ± 2.33	1.25 ± 0.56	13.22 ± 4.05	2.16 ± 0.75
Control group (*n* = 108)	8.87 ± 1.89	1.38 ± 0.48	16.13 ± 5.42	3.22 ± 1.10
*t* value	0.48	− 1.83	− 4.46	− 8.26
*P* value	0.629	0.069	< 0.001	< 0.001

*MIE*, minimally invasive esophagectomy

*TUGT*, timed up and go test

The t-ECRP was even more effective than routine care in improving physical function recovery as measured by the TUGT (s) and frailty score. As summarized in Table 4, before the intervention (the day of admission), no significant differences in baseline physical functions between the two groups were observed (*P* > 0.05). After the t-ECRP intervention (the day of discharge), the mean length of time for TUGT (s) (13.22) and a score of frailty (2.16) in the IG were lower than those in the CG (16.13 and 3.22), indicating that the physical function recovery in the IG was significantly better than that in the CG (*P* < 0.001).

After the t-ECRP intervention, except for the dimension of expected support, the total scores of RHD (*P* < 0.001), the dimension of physical status (*P* < 0.001), and adaptive ability (*P* = 0.001) were significantly higher in the IG than those in the CG, as shown in Table 5. Likewise, compared with the CG, patients in the IG presented a significantly shorter time length of postoperative hospital stay (9.08 ± 3.48 days vs 12.14 ± 4.05, *t* =  − 5.94, *P* < 0.001).

**Table 5:** The level of RHD of patients with MIE in intervention and control groups on the day of discharge

Dimensions of RHD	Intervention group (*n* = 107)	Control group (*n* = 108)	*t* value	*P* value
Physical status	8.48 ± 1.45	7.57 ± 1.82	4.06	< 0.001
Adaptive ability	8.82 ± 1.50	8.01 ± 2.05	3.31	0.001
Expected support	9.05 ± 2.85	8.35 ± 2.70	1.85	0.066
Total	8.92 ± 1.42	7.86 ± 1.79	4.81	< 0.001

*RHD*, readiness for hospital discharge

*MIE*, minimally invasive esophagectomy

## Discussion

Esophagectomy has been identified as a particularly complex surgical procedure due to documented high levels of perioperative morbidity and mortality [26]. Advances in perioperative management concepts and medical technology have been proposed to be able to reduce surgical risk and perioperative morbidity and mortality, thus improving surgical short- and long-term outcomes [27–29]. According to the components of ERAS guidelines, early and structured mobilization is an essential factor for accelerated recovery, and there is a strong relationship between physical activity and quality of life generally [30]. Ambulating early not only can prevent complications associated with bed rest and maintain muscle function but also empowers patients to play an active role in their rehabilitation after surgery [7]. Therefore, an early and tailored daily perioperative rehabilitation plan for patients with MIE should be formulated by the involvement of thoracic surgeons, nurses, and physiotherapists.

Cardiopulmonary fitness and physical function are key determinants of fitness for major thoracic surgery [31]. One strength of our study is preoperative rehabilitation, which included SCT and IMT and was a part of the t-ECRP intervention. “Pre-rehabilitation before the operation can accelerate recovery after operation.” This is the philosophy of our team in the implementation of ERAS. The preoperative pre-rehabilitation strategy includes psychological counseling, nutritional supplementation, physical exercise, and respiratory optimization. Studies have shown that physical exercise programs involving both aerobic and strengthening activities reduce depression, anxiety, and fatigue, and improve the quality of life [32, 33]. One evidence-based scoping review [34] evaluating the possible beneficial effects of preoperative exercise therapy on surgery showed that the preoperative exercise programs could increase exercise capacity and physical fitness, preserve pulmonary function, reduce the incidence of postoperative complications, and decrease the length of hospital stay. Although some studies [7] suggest that the preoperative rehabilitation program should be implemented at least for 4 weeks, there is limited data about the general consensus or clear practical guidance regarding exercise methods and exercise time norms for esophagectomy.

This randomized clinical trial provided evidence that t-ECRP, involving pre-rehabilitation and early postoperative activity, promoted effective recovery of bowel function and physical function in patients undergoing MIE. TUGT is a standard method to observe a patient’s motor functions and daily activities and is an important index to evaluate a patient’s prognosis [35]. Although the physical fitness of EC patients was affected to a certain extent due to the operation, analysis of this study showed that the time length of TUGT in the IG (13.22±4.05) was significantly shorter than that in the CG (16.13±5.42) after discharge. The frailty scores ranged from 1 to 4, and there were significant statistical differences between the two groups. It should be noted that 32.09% of patients were in frailty states (three or more scores) and 56.28% in pre-frailty states (one or two scores) after MIE, suggesting a requirement for special attention.

RHD is a patient’s self-perception of patients about whether they are ready to be discharged. It is related to medical satisfaction and safety after discharge. Studies [36, 37] have shown that the higher RHD, the stronger ability to cope with health challenges after discharge. In this study, the RHD of patients after MIE was at a medium level. Given that physical recovery is closely related to a patient’s self-feeling and self-care ability in life when discharged from hospital, an improvement in RHD is thus hypothesized to be a potential secondary benefit of this program. Furthermore, t-ECRP is beneficial to the enhancement of RHD and shortening of the postoperative hospital stay. Surprisingly, in this trial, the findings showed that the postoperative hospital stay was approximately 3 days shorter in the t-ECRP group (9.08±3.48 days) than that in the routine care group (12.14±4.05 days). One systematic review consisting of 26 studies showed that early enteral nutrition could promote intestinal function recovery and shorten the time of postoperative hospital stay for patients undergoing gastrointestinal surgery [38]. This reduced postoperative hospital stay was likely the result of early flatus and bowel movement after surgery, which shortened the fasting time of patients, and achieved the purpose of early oral intake, nutrition improvement, and fast postoperative recovery.

Maximizing the patient’s subjective initiative in disease management during the perioperative rehabilitation process is very important. Therefore, before the program is formulated, researchers need to explain the concept of ERAS and the significance of early activities to patients and discuss pre- and postoperative rehabilitation types and target amount together. Moreover, positive encouragement should be given when the target is completed, and adjustment of rehabilitation plan should be conducted based on cause analysis of researcher and patient, when the goal is not completed.

In our study, some efforts were also made to provide the foundation and guarantee for the implementation of t-ECRP, such as adequate analgesia management and extubation as soon as possible. Previous data [39] showed that adequate pain management accelerated recovery of bowel function, increased patient mobility, decreased hospital stay, and optimized patient outcomes. Therefore, painlessness is a prerequisite for early postoperative activities. In our study, multimodal analgesia and individualized analgesia programs were used to control the patient’s pain to make it less than 3 points (visual analogue scoring). Besides, tubes on the patient’s body can hinder postoperative activities, especially the urinary tube and gastric tube. Hence, our team adhered to the concept of early extubation as soon as possible after evaluation by the research team to facilitate activities [40].

### Strengths and limitations

The advantage of this study lies in its emphasis on the subjective initiative of the patients in rehabilitation and the establishment of a professional multidisciplinary team to ensure patient safety. This study also had some notable limitations. First, due to the limited preoperative time, the time of preoperative rehabilitation in this study was relatively short (approximately 7–10 days), which might not be able to offer full improvement in fitness. Second, in this study research staff were aware of the interventions and randomization results. Despite all efforts to maintain blinding, we could not implement a double-blind method owing to the nature of the interventional research. Third, due to the limitations of the research conditions, we could not evaluate patients’ electrophysiological indicators to reflect the improvement of physical function, which is an important research field of rehabilitation medicine.

## Conclusion

In conclusion, the current study showed that the t-ECRP, a nurse-led and three-staged procedure, was practical and feasible in accelerating bowel and physical function recovery for patients receiving MIE based on the context of ERAS. Besides, t-ECRP can also improve patients’ RHD and shorten postoperative hospital stay, which may enhance patients’ medical experience and hospital operation efficiency. Clinical nurses play a key role in patients’ perioperative enhanced recovery. The results of this research provide support for the formulation of quantitative, detailed, and individualized early activity plans for patients based on multidisciplinary collaboration.

## Data Availability

This article is distributed under the terms of the Creative Commons Attribution 4.0 International License, which permits unrestricted use, distribution, and reproduction in any medium, provided you give appropriate credit to the original author(s) and the source, provide a link to the Creative Commons license, and indicate if changes were made.

## Code availability

N/A
